# The relationship between self-oriented perfectionism and athlete burnout: a longitudinal study

**DOI:** 10.3389/fpsyg.2025.1656816

**Published:** 2025-10-16

**Authors:** Yuantai Fu, Xingyi Li, Junjun Sun, Caixia Li, Yang Peng, Fuxin Hong, Jianhua Pan

**Affiliations:** ^1^Department of Marine Sports, Pukyong National University, Busan, Republic of Korea; ^2^Department of Sport and Health, Shinhan University, Uijeongbu-si, Republic of Korea; ^3^School of Foreign Language, Shandong Vocational and Technical University of International Studies, Rizhao, Shandong, China; ^4^Department of Physical Education, Taiyuan Institute of Technology, Taiyuan, Shanxi, China; ^5^College of Physical Education, Guizhou Normal University, Guiyang, Guizhou, China; ^6^College of Physical Education, Chongqing University, Chongqing, China

**Keywords:** self-oriented perfectionism, athlete burnout, loneliness, random-intercept cross-lagged panel model, longitudinal study

## Abstract

**Background:**

Athlete burnout is a critical psychological concern, and perfectionism—characterized by excessively high standards and self-critical tendencies—may play a role in its onset. However, prior findings on the link between self-oriented perfectionism (SOP) and burnout remain inconsistent, and the mechanisms underlying this relationship are not fully understood.

**Methods:**

This longitudinal study employed a Random-Intercept Cross-Lagged Panel Model (RI-CLPM) to examine reciprocal associations between SOP and athlete burnout, as well as the mediating role of loneliness. A total of 422 athletes (*M*_age_ = 19.74, SD = 1.77) completed three waves of assessments between June 2024 and June 2025.

**Results:**

SOP significantly predicted subsequent athlete burnout (SOP_T1 → AB_T2: *β* = 0.355, *p* < 0.001; SOP_T2 → AB_T3: *β* = 0.374, *p* < 0.001). Loneliness emerged as a significant mediator: higher SOP predicted increased loneliness (*β* = 0.376, *p* < 0.001), which in turn was associated with greater burnout (*β* = 0.265, *p* < 0.01). The indirect effect was significant (*β* = 0.099, 95% CI [0.033, 0.209]). Notably, no evidence supported a reverse pathway from burnout to subsequent SOP.

**Discussion:**

SOP is a longitudinal risk factor for athlete burnout, partly through its contribution to heightened loneliness. Preventive interventions that enhance social and coach support, alongside resilience-building strategies such as mindfulness, may help reduce vulnerability to burnout in athletes.

## 1 Introduction

Athlete burnout refers to a psychological and physiological condition that arises when athletes experience a persistent imbalance between personal resources and environmental demands due to prolonged high-intensity training and competitive pressure (Gustafsson et al., 2017). This imbalance results in sustained psychological and physiological stress responses, which gradually evolve into symptoms of emotional and physical exhaustion, diminished sense of accomplishment, and negative evaluations of sport, ultimately leading to a dysfunctional state characterized by impaired psychological. Athlete burnout exerts profound effects on individual development (Glandorf et al., 2025; Olsson et al., 2025), professional careers (Lin et al., 2022), and the broader sports ecology (Pacewicz et al., 2019). Due to constant exposure to competitive pressure, training loads, injury risks, and societal expectations, the incidence of burnout among athletes is significantly higher than that of the general population (Gao and Wang, 2024; Granz et al., 2019). The past two decades have witnessed a substantial rise in athlete burnout (Madigan et al., 2022). In particular, the severity of reduced athletic accomplishment and sport devaluation among athletes has increased considerably (Madigan et al., 2022).

In recent years, researchers have elucidated the role of perfectionism in athletes’ mental health (McLoughlin et al., 2024; Shi, 2023; Watson et al., 2023; Hill et al., 2018). Perfectionism is typically conceptualized as a multidimensional personality trait that can be categorized into three primary dimensions (Hewitt and Flett, 1991). Self-oriented perfectionism refers to individuals’ tendency to set excessively high and often unrealistic standards for themselves, accompanied by harsh self-criticism and self-evaluation. Its core characteristics include maintaining extremely high expectations for personal performance, difficulty tolerating personal errors or imperfections, engaging in continuous self-monitoring, and experiencing persistent dissatisfaction even when achievements are attained (Stoeber et al., 2009). Other-oriented perfectionism manifests as imposing unrealistic high demands on others’ behaviors, capabilities, and achievements, while evaluating them according to stringent standards. This tendency is prone to generating interpersonal conflicts and strained relationships, as individuals often respond with criticism and disappointment when others fail to meet their expectations (Stricker et al., 2019). Finally, socially prescribed perfectionism refers to individuals’ perception of externally imposed high standards, which they internalize as benchmarks for self-worth evaluation. Individuals exhibiting this type typically believe that recognition can only be obtained through meeting others’ or societal expectations, consequently experiencing chronic stress and being more susceptible to psychological distress such as low self-esteem, anxiety, social withdrawal, and depression (Stoeber et al., 2009). These three perfectionism indices represent different psychological mechanisms, and as independent constructs at a higher-order level, they can be employed either independently or in combination (Soenens et al., 2005).

Multiple studies have confirmed the link between perfectionism and athlete burnout (Appleton et al., 2009; Olsson et al., 2022b; Hill et al., 2008, 2018). Despite this established connection, the specific nature of the relationship between self-oriented perfectionism (SOP) and athlete burnout remains complex, with studies reporting inconsistent patterns of association. Some researchers have argued that SOP is associated with higher levels of intrinsic motivation, thereby exhibiting a negative correlation with burnout (Chang et al., 2015; Labrague, 2024). Conversely, other research suggests that SOP demonstrates no significant association with burnout or exhibits a negative correlation (Moore et al., 2018; Navaitienė and Danilovienė, 2017; Stoeber et al., 2008; Stoeber and Becker, 2008). This inconsistency indicates that the pathway from SOP to burnout does not operate through direct effects, but rather may exert its influence through mediating effects of other psychological variables. Loneliness represents one such potential mediating factor that has not yet been sufficiently explored (Harper et al., 2020). Loneliness is defined as the subjective distressing experience that occurs when individuals perceive a discrepancy between their ideal and actual social relationships (Cramer and Barry, 1999). Research demonstrates that loneliness is not only closely associated with various negative emotions and mental health problems, but may also exacerbate athletes’ stress responses and diminish their recovery capacity (Brown et al., 2018; Kang et al., 2024). In recent years, loneliness has been increasingly recognized as a significant mental health concern facing athletes (Jackman et al., 2024). Based on this foundation, the present study employs Self-Determination Theory and the Perfectionism Social Disconnection Model as theoretical frameworks to investigate in depth the mediating mechanisms of loneliness in the aforementioned relationships.

Although existing literature has provided preliminary attention to the impact of perfectionism on athletes’ mental health, considerable limitations remain. First, the underlying mechanisms between SOP and burnout have yet to be systematically and thoroughly explored from a theoretical perspective. Second, most of the studies in the current literature have employed cross-sectional designs (Hill et al., 2008; Kang and Gong, 2024), lacking longitudinal examinations of the relationship between athlete perfectionism and professional burnout. Third, among the limited longitudinal studies (Jowett et al., 2016; Xu et al., 2024), the cross-lagged panel model (CLPM) is predominantly utilized, which can conflate interpersonal and intra-individual effects, thereby potentially resulting in overestimated or even reversed findings (Hamaker et al., 2015). Moreover, existing longitudinal research has not sufficiently investigated the potential mediating mechanisms underlying the influence of perfectionism on athlete burnout, thereby constraining substantive mechanism exploration to cross-sectional study designs. These limitations hinder a deeper understanding of the dynamic causal relationship between multidimensional perfectionism and athlete burnout.

To address this gap, the present study employs the Random-Intercept Cross-Lagged Panel Model (RI-CLPM) to distinguish intra-individual effects from interpersonal effects. By utilizing three-wave longitudinal data, this study revealed the potential pathway through which self-oriented perfectionism elicits loneliness in athletes, subsequently leading to occupational burnout, subsequently leading to professional burnout. This approach offers novel insights into the dynamic interplay between SOP and professional burnout within the context of competitive sports.

## 2 Literature review

### 2.1 The relationship between self-directed perfectionism and athlete burnout

Existing research regarding SOP and athlete occupational burnout has found that there is a direct association between SOP and increased occupational burnout (Hill et al., 2008; Hill and Appleton, 2011; Luo et al., 2016; Martin et al., 2022). When individuals with SOP perceive themselves as failing to meet high standards, they engage in harsh self-criticism, which undermines their self-esteem and leads to emotional exhaustion and cynicism (Luo et al., 2016; Minichiello et al., 2024). This persistent self-denigration and negative evaluation can result in feelings of frustration and helplessness, gradually diminishing enthusiasm toward their work. Additionally, the fear of failure may prompt individuals to procrastinate or avoid tasks, as they fear being unable to perform them perfectly. Such avoidant behavior ultimately increases stress and feelings of incompetence, further exacerbating burnout (Cho and Lee, 2022; Moroz and Dunkley, 2019). In competitive sports, high self-standards often trap athletes in a “must succeed, cannot fail” cognitive mindset, thereby amplifying negative emotional experiences. When chronically in this state, athletes may still feel that their performance is “not good enough” even after achieving excellent results, perceiving goals as difficult to attain and gradually losing satisfaction with their performance and sense of worth, ultimately leading to burnout (Bum et al., 2017). Research by Olsson et al. indicates that after controlling for various dimensions of sport performance perfectionism, self-oriented perfectionism still demonstrates a significant positive correlation with athlete burnout (Olsson et al., 2022b). Additionally, self-oriented perfectionism positively correlates with autonomous motivation, as athletes often pursue high standards based on personal values and intrinsic interest. However, when athletes highly internalize perfectionistic standards while the external motivational climate conflicts with their intrinsic goals, significant psychological conflict may emerge, subsequently inducing burnout (Madigan et al., 2016). However, some studies have also found that SOP may produce positive effects, including higher productivity, success, self-confidence, positive emotions, and intrinsic motivation (Moore et al., 2018; Navaitienė and Danilovienė, 2017; Stoeber et al., 2008; Stoeber and Becker, 2008). Research indicates that the intrinsic drive and adherence to high standards associated with SOP can strengthen athletes’ engagement and value identification with their sport, thereby buffering against the development of cynicism and making SOP a protective factor in certain contexts (Yang et al., 2023). These contradictory findings may stem from critical methodological limitations in existing research. First, the vast majority of studies employ cross-sectional designs, which can only capture associations at a single time point and cannot establish the temporal precedence required for causal inference (Savitz and Wellenius, 2023). In fact, bidirectional relationships may exist between SOP and burnout: perfectionism may lead to burnout, while burnout may, in turn, exacerbate perfectionistic tendencies as athletes strive to regain a sense of control and competence. Second, traditional longitudinal analytical approaches (such as CLPM) cannot distinguish between-person differences from within-person changes over time (Hamaker et al., 2015). For example, finding that athletes with higher levels of SOP also report higher burnout (between-person association) does not necessarily imply that increases in SOP over time within a given athlete will lead to increases in that athlete’s burnout (within-person association). This distinction is crucial because interventions typically aim to change individuals’ states over time, rather than merely screening for people with different traits.

Few studies have examined the reverse association, and no research to date has explored burnout as a potential antecedent of self-oriented perfectionism. Nevertheless, some studies have investigated the effects of burnout on constructs conceptually related to or encompassed by perfectionism. Cross-sectional studies have suggested that the occurrence of professional burnout is often accompanied by high levels of self-criticism and negative affect, a pattern that is particularly evident in high-stress work environments (Ferrari et al., 2018; Kim, 2024). Moreover, research by Gustafsson et al. (2017) and others has found that adolescent athletes with a high fear of failure are more susceptible to psychological stress and professional burnout. In the nursing field, studies have further revealed that diminished work motivation resulting from burnout can reduce nurses’ work engagement and intrinsic motivation, subsequently leading to decreased caregiving behaviors (Labrague, 2024; Nabizadeh-Gharghozar et al., 2020). Meanwhile, some theoretical perspectives suggest that burnout may exert prospective influences on perfectionism. According to compensatory control theory, humans possess an inherent need to maintain a sense of order and control (Kay et al., 2009). The emotional exhaustion and sense of loss of control induced by burnout undermine this perception. To compensate for the deficit in perceived control, some individuals may elevate their perfectionistic standards, reducing failure risk and restoring psychological mastery through more stringent self-demands. Second, from the perspective of cognitive dissonance theory, individuals pursue psychological consistency, and when conflicts arise between their behaviors, emotional states, and self-concept, psychological discomfort—namely, cognitive dissonance—emerges (Elliot and Devine, 1994). Athletes whose core self-concept centers on high achievement experience self-inconsistency when burnout leads to performance decline. To alleviate this dissonance, they may intensify perfectionistic beliefs and raise standards to maintain their high-achievement identity and restore psychological coherence. These findings provide both theoretical and empirical foundations for understanding the potential associations between perfectionism and burnout. These findings provide both theoretical and empirical foundations for understanding the potential links between perfectionism and burnout.

Based on the aforementioned theories and literature, we posit that a bidirectional relationship may exist between SOP and athlete burnout, warranting further investigation. Exploring this relationship could enable future research to develop a more comprehensive understanding of the psychological factors influencing athlete burnout. This endeavor holds substantial practical significance for promoting athlete well-being, preventing burnout, and potentially enhancing performance by fostering healthier approaches to striving for excellence.

### 2.2 The mediating effect of loneliness

Loneliness is a complex psychological experience that arises when individuals perceive a discrepancy between the quality and quantity of their social relationships and their desired expectations (Cramer and Barry, 1999). Unlike objective social isolation, loneliness is a subjective perception of a deficiency in social interactions (Ge et al., 2017). It emerges when there is a mismatch between desired intimate relationships, group belonging, or social support and what is actually attained (Coplan et al., 2019; Long and Averill, 2003).

Research has demonstrated that athletes with SOP experience feelings of inadequacy and engage in self-criticism when they fail to meet their self-imposed high standards, subsequently leading to social withdrawal and the development of loneliness (Jackman et al., 2024; Donachie et al., 2019). Moreover, perfectionists not only impose stringent demands on themselves but may also hold excessively high expectations for others. Such behavior can provoke interpersonal conflicts or make others perceive them as difficult to approach, thereby undermining the formation of close relationships and increasing loneliness (Li et al., 2021; Visvalingam et al., 2024). Loneliness, as a negative emotional experience, can lead to a lack of social support and a diminished sense of belonging, thereby increasing the risk of emotional exhaustion (Pacewicz and Smith, 2022; Roberts and Krueger, 2021). A cross-sectional study involving 234 athletes demonstrated that those with greater social support and connections exhibited lower levels of athletic identity disintegration and higher levels of psychological well-being (Roberts and Krueger, 2021). Therefore, when athletes feel isolated and unsupported within their teams, they may develop negative perceptions of their teammates and coaches, gradually becoming indifferent and detached. This process of depersonalization not only undermines team cohesion but also further exacerbates athletes’ burnout levels (Anderson and Dixon, 2019; Chen et al., 2024; DeFreese and Smith, 2014; Gano-Overway and Peterson, 2023).

The Perfectionism Social Disconnection Model (PSDM) integrates attachment theory, self-psychology, and negative emotional state theory, proposing that perfectionism is not merely a personal trait but also serves crucial interpersonal functions in social interactions (Chen et al., 2015). Specifically, perfectionists often pursue excellence to fulfill their needs for belonging and self-esteem, yet such efforts frequently prove counterproductive (Hewitt et al., 2006). At the behavioral level, perfectionists typically exhibit heightened sensitivity to rejection, hostility and aggressive tendencies, self-concealment behaviors, and excessive reassurance-seeking from others. These behaviors not only increase tension and friction in interpersonal interactions but also gradually erode their emotional connections with others. In competitive sports contexts, athletes with self-oriented perfectionism may appear goal-oriented and highly motivated on the surface, yet their behaviors are often driven by the belief that “one must perform perfectly” (Stoeber et al., 2021). This tendency leads them to avoid displaying vulnerability during training and competition while being reluctant to actively seek help, gradually developing an interpersonal strategy characterized by intimacy avoidance and excessive self-protection. Over time, this highly defensive and controlling approach to interpersonal interactions undermines emotional intimacy and trust with coaches, teammates, and even friends, resulting in increasingly superficial relationships and elevated subjective loneliness (Olsson et al., 2022b). Therefore, within the PSDM framework, self-oriented perfectionism disrupts individuals’ social interaction patterns, weakens their social connections, and subsequently triggers loneliness as a psychological manifestation of social disconnection. Furthermore, the PSDM not only reveals the existence of social disconnection but also emphasizes its profound psychological consequences, with interpersonal disconnection being conceptualized as an antecedent factor for various forms of psychological distress, including depression, anxiety, and emotional exhaustion. In athletic contexts, the absence of social support—a crucial stress buffering mechanism—renders athletes more susceptible to emotional exhaustion; the lack of encouragement and validation from peers undermines their sense of accomplishment; and when individuals perceive themselves as unaccepted by the collective, they may develop feelings of alienation or even cynicism toward the sport they once loved, manifesting as “sport devaluation.” These factors constitute the core dimensions of athlete burnout (Markati et al., 2019; Gray et al., 2024). In other words, from the PSDM perspective, loneliness serves not merely as an interpersonal byproduct of self-oriented perfectionism but also functions as a crucial mediating mechanism between perfectionism and burnout. It reflects both the adaptive costs of perfectionism at the social level and reveals the social–emotional pathway through which athlete burnout develops, thereby providing an important theoretical anchor for understanding this psychological phenomenon. From the perspective of Self-Determination Theory (SDT), the mechanism through which athletes’ self-oriented perfectionism leads to sport burnout by increasing loneliness can be explained through basic psychological need frustration. SDT posits that humans universally possess three basic psychological needs: autonomy (feeling that behavior is voluntary and self-chosen), competence (feeling capable of mastering tasks and producing effectiveness), and relatedness (feeling connected to and belonging with others) (Adams et al., 2017). When these needs are satisfied, individuals experience higher levels of intrinsic motivation and psychological well-being; conversely, when these needs are chronically frustrated, psychological distress and maladjustment are likely to occur (Deci and Ryan, 2008). Self-oriented perfectionistic athletes often fear exposing their flaws and worry that imperfection will lead to rejection or loss of respect (Lundqvist et al., 2024). Due to these concerns, they tend to conceal their true feelings and difficulties, avoid seeking social support, and view help-seeking as a sign of weakness (Cosh et al., 2024). Such avoidance behaviors impede their ability to establish authentic and deep interpersonal connections with coaches, teammates, and family members, undermining the satisfaction of relatedness and ultimately triggering emotional alienation and loneliness. Loneliness itself is a chronic psychological stressor (Beutel et al., 2017) that depletes additional psychological resources and reduces athletes’ capacity to cope with training and competition pressures. Furthermore, the absence of meaningful interpersonal connections deprives sport achievements of their value for sharing and social recognition, causing training and competition to gradually become isolated processes of self-validation rather than sources of joy and accomplishment. This undermines autonomy experiences, further reduces intrinsic motivation, and thereby increases the risk of sport burnout (Pacewicz et al., 2025).

Based on the aforementioned research and theories, we hypothesized that self-oriented perfectionism would indirectly predict athlete burnout through loneliness. However, previous studies have predominantly regarded loneliness as a psychological issue affecting adolescent or elderly populations (Bessaha et al., 2020; Hemberg et al., 2022). This study demonstrates the triggering mechanisms of loneliness within competitive sports contexts, providing cross-contextual evidence for the application of loneliness theories in high-pressure performance environments among athletes.

### 2.3 The present study

This study employed a RI-CLPM to explore the relationship between SOP and professional burnout from both within-person and interpersonal perspectives, as well as the mediating role of loneliness. We proposed two related hypotheses: (1) SOP and professional burnout will significantly predict each other. (2) SOP and athlete burnout can indirectly predict each other through loneliness.

## 3 Methods

### 3.1 Participants and procedures

This study was a longitudinal investigation that received ethical approval from the local ethics review committee in accordance with established guidelines (Ethics Approval Number: SVTUIS20240404). A stratified sampling method was employed to recruit participants from six randomly selected universities among 26 higher education institutions located in southern provinces of China. Data collection was conducted in three waves from Time 1 (T1) to Time 3 (T3), with six-month intervals between each wave. As the data collection was part of a larger research project, participants completed a total of nine self-report instruments, of which three were relevant to the present study. Ultimately, 473 athletes participated in the study. The valid sample size was 452 at Time 1 (June 2024), 435 at Time 2 (December 2024), and 422 at Time 3 (June 2025). Participants who completed all three measurements of the key variables and provided valid responses at Time 1 were included in the final analysis. Thus, the final analytical sample consisted of 422 participants (those who continuously completed the questionnaires from Time 1 to Time 3). The age of the athletes ranged from 17 to 25 years (*M*_age_ = 19.74, SD = 1.77), with 60.6% being male and 39.4% female. Their athletic career length ranged from 5 to 13 years (*M* = 8.33, SD = 2.01).

Prior to the commencement of the study, informed consent was obtained from all participants, ensuring that their participation was entirely voluntary and that all data were fully anonymized (For minor participants aged 17, we obtained informed consent forms signed by the participants themselves while simultaneously providing their legal guardians with detailed explanations of the research objectives, procedures, potential risks, and the right to withdraw, and securing written informed consent from them as well). Data collection at all time points was conducted through on-site completion of paper-based questionnaires. Research assistants clearly explained the procedure for completing the questionnaires and the purpose of the study to the athletes. Each survey session lasted approximately 40 min, and all participants were informed that they could withdraw from the study at any time without any negative consequences. The minimum sample size for this study was determined based on a 1:5 item-response ratio, indicating that at least 190 cases were required for the 38 items assessed (Qu, 2007). Little’s MCAR test was employed to evaluate whether data were missing completely at random (MCAR) across all variables at each measurement point (Little, 1988). The test results were χ^2^ = 24.236, df = 15, *p* = 0.061. This indicates that the data were missing completely at random, allowing the use of Full Information Maximum Likelihood (FIML) estimation for subsequent model analysis.

### 3.2 Measures

All measurement instruments used in this study were translated versions based on existing scales. The Chinese versions of these scales have demonstrated satisfactory psychometric properties in previous research involving Chinese populations (Guo et al., 2022; Liu et al., 2022; Ip et al., 2024) and exhibited good reliability and validity in the present study.

#### 3.2.1 Self-oriented perfectionism

Self-oriented perfectionism was measured using the self-oriented perfectionism subscale of the Multidimensional Perfectionism Scale (MPS; Hewitt and Flett, 1991). The SOP subscale consists of 15 items that measure individuals’ personal standards and expectations concerning their own performance and behavior (e.g., “I set goals that strive for perfection”). In the present study, the Cronbach’s alpha coefficients for the SOP subscale across T1, T2, and T3 were 0.930, 0.950, and 0.949, respectively.

#### 3.2.2 Loneliness

Loneliness was measured using the UCLA Loneliness Scale (Hays and DiMatteo, 1987), which is a shortened version of the original UCLA Loneliness Scale (ULS-20). In the present study, the Cronbach’s alpha coefficients for the ULS-8 across T1, T2, and T3 were 0.901, 0.894, and 0.897, respectively.

#### 3.2.3 Athlete burnout

Athlete burnout was measured using the Athlete Burnout Questionnaire (ABQ) developed by Raedeke and Smith (2001). In the present study, the Cronbach’s alpha coefficients for the ABQ across T1, T2, and T3 were 0.877, 0.888, and 0.893, respectively.

### 3.3 Data analysis

First, correlation analyses and descriptive statistics among variables were conducted using SPSS 24.0. To capture each participant’s stable trait level, the intraclass correlation coefficient (ICC) was calculated for each variable. Second, confirmatory factor analysis (CFA) was performed to test longitudinal measurement invariance across the three time points. According to the criteria suggested by Chen (2007), measurement invariance is established if model fit does not significantly deteriorate when imposing equality constraints. Third, two RI-CLPM were constructed to test Hypothesis 1 and Hypothesis 2. Compared to traditional Cross-Lagged Panel Models (CLPM), RI-CLPM allows for the separation of stable between-person differences from within-person changes over time, thereby providing clearer causal inferences. Given that the data exhibited a non-normal distribution, the Maximum Likelihood Robust (MLR) estimation method was employed to obtain more accurate parameter estimates. After comparing the baseline model (in which autoregressive and cross-lagged paths were constrained to be equal over time) with alternative models, the best-fitting model was selected. The significance, magnitude, and direction of the mediating effect of loneliness were examined using bootstrapping analysis with 10,000 bootstrap samples.

Given that gender role expectations in sport contexts may moderate the longitudinal relationships among these psychological variables, thereby influencing within-individual causal dynamics (Amemiya and Sakairi, 2020; Chalabaev et al., 2013; Lainas and Cho, 2017), neglecting such potential differences could lead to model interpretation bias and even misleading conclusions. To ensure the robustness of our research findings, we conducted multi-group comparisons to examine the invariance of model paths across different gender groups.

## 4 Results

### 4.1 Descriptive statistics

Table 1 presents the correlations, means, and standard deviations of all variables. The ICCs for SOP, loneliness, and athlete burnout were 0.595, 0.725, and 0.605, respectively. The within-person variance of all variables exceeded 10%, indicating the suitability of employing the RI-CLPM for measurement (Hamaker et al., 2015).

**Table 1:** Descriptive statistics and correlations for variables.

	1	2	3	4	5	6	7	8	9	*M*	SD
1. T1 SOP	1									3.247	0.941
2. T2 SOP	0.734^**^	1								3.140	0.965
3. T3 SOP	0.535^**^	0.563^**^	1							2.973	1.089
4. T1 AB	0.272^**^	0.214^**^	0.102^*^	1						2.717	0.828
5. T2 AB	0.411^**^	0.333^**^	0.163^**^	0.578^**^	1					2.660	0.857
6. T3 AB	0.389^**^	0.424^**^	0.138^**^	0.534^**^	0.689^**^	1				2.623	0.859
7. T1 LON	0.363^**^	0.364^**^	0.220^**^	0.274^**^	0.361^**^	0.336^**^	1			2.427	0.678
8. T2 LON	0.410^**^	0.400^**^	0.171^**^	0.288^**^	0.407^**^	0.418^**^	0.753^**^	1		2.411	0.668
9. T3 LON	0.294^**^	0.294^**^	0.162^**^	0.189^**^	0.329^**^	0.311^**^	0.686^**^	0.742^**^	1	2.342	0.673

SOP, Self-Oriented Perfectionism; LON, Loneliness; AB, Athlete burnout; M, Mean; SD, Standard Deviation. *p < 0.05, **p < 0.01, ***p < 0.001.

### 4.2 Longitudinal measurement invariance

Table 2 presents the longitudinal measurement invariance of all variables. The decline in CFI for each variable was less than 0.01, and the increase in RMSEA did not exceed 0.015, demonstrating strong longitudinal invariance. This indicates that the measurement instruments exhibit high structural consistency and reasonable comparability across different time points (Chen, 2007).

**Table 2:** Fit indices for measurement invariance of main variables.

Variables	Model tested	CFI	TLI	RMSEA	SRMR	ΔCFI	ΔRMSEA	ΔSRMR
	Configural invariance	0.963	0.959	0.033	0.030	–	–	–
Self-oriented perfectionism	Metric invariance	0.962	0.959	0.033	0.035	0.001	0.000	0.003
	Scalar invariance	0.959	0.957	0.034	0.038	0.004	0.000	0.002
	Configural invariance	0.952	0.941	0.053	0.032	–	–	–
Loneliness	Metric invariance	0.951	0.944	0.051	0.037	0.001	0.002	0.005
	Scalar invariance	0.949	0.945	0.051	0.040	0.003	0.002	0.002
	Configural invariance	0.970	0.967	0.024	0.037	–	–	–
Athlete burnout	Metric invariance	0.970	0.968	0.024	0.039	0.000	0.000	0.006
	Scalar invariance	0.968	0.967	0.024	0.043	0.002	0.000	0.006

CFI, Comparative Fit Index; RMSEA, Root Mean Square Error of Approximation.

### 4.3 The results of random intercept cross-lagged panel models

As shown in Table 3, the constrained RI-CLPM (with equality constraints on autoregressive and cross-lagged paths) demonstrated comparable fit to the unconstrained model, with no significant decrement in model performance. Considering interpretability, Models M1a and M2a were selected as the final models.

**Table 3:** Model fit of RI-CLPMs.

Models	χ^2^*(df)*	CFI	SRMR	RMSEA [90% CI]	∆CFI	∆RMSEA
Model 1						
M1a	37.788 (21)	0.992	0.022	0.042 [0.019, 0.063]	–	–
M1b	39.385 (24)	0.992	0.023	0.038 [0.014, 0.058]	0.000	0.004
M1c	58.601 (27)	0.984	0.035	0.051 [0.033, 0.069]	0.008	0.009
Model 2						
M2a	22.018 (13)	0.992	0.024	0.039 [0.000, 0.067]	–	–
M2b	30.076 (15)	0.987	0.042	0.047 [0.022, 0.072]	0.005	0.018
M2c	22.762 (15)	0.993	0.035	0.034 [0.020, 0.066]	0.001	0.005

M1a/M2a = Fully unconstrained RI-CLPM; M1b/M2b = RI-CLPM with time invariance constraints on the autoregressive stabilities; M1c/M2c = RI-CLPM with time invariance constraints on the cross-lagged effects. RMSEA, Root Mean Square Error of Approximation; CI, Confidence Interval; SRMR, Standardized Root Mean Square Residual.

**Figure 1:**
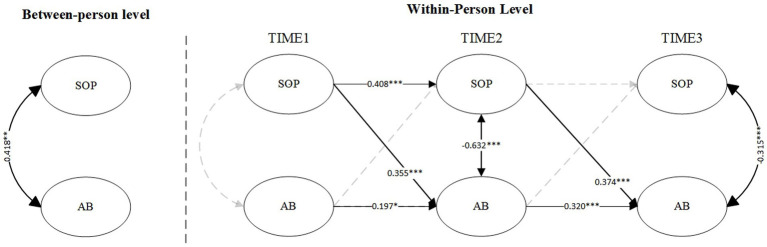
RI-CLPM for self-oriented perfectionism and athlete burnout (Model 1). SOP, Self-Oriented Perfectionism; AB, Athlete burnout. Solid lines represent the significant paths, dashed lines represent the non-significant paths. *p < 0.05, **p < 0.01, ***p < 0.001.

Figure 1 displays the RI-CLPM results for SOP and athlete burnout. At the between-person level, SOP exhibited a significant positive association with athlete burnout (β = 0.418, *p* < 0.001), representing a moderate-to-large effect size according to Cohen’s conventions. At the within-person level, the paths from SOP at Time 1 (T1) to athlete burnout at Time 2 (T2) and from SOP at Time 2 (T2) to athlete burnout at Time 3 (T3) were significant (SOP_T1 to AB_T2: β = 0.355, *p* < 0.001; SOP_T2 to AB_T3: β = 0.374, *p* < 0.001), both indicating moderate effect sizes. However, the reverse paths were not significant. These cross-lagged coefficients (SOP_T1 → AB_T2: β = 0.355; SOP_T2 → AB_T3: β = 0.374) suggest that SOP consistently accounts for approximately 12.6 and 14.0% of the variance in subsequent athlete burnout, respectively. This consistent predictive strength across different time points represents a meaningful and stable relationship that may be sufficiently robust to inform targeted interventions in sport settings.

**Figure 2:**
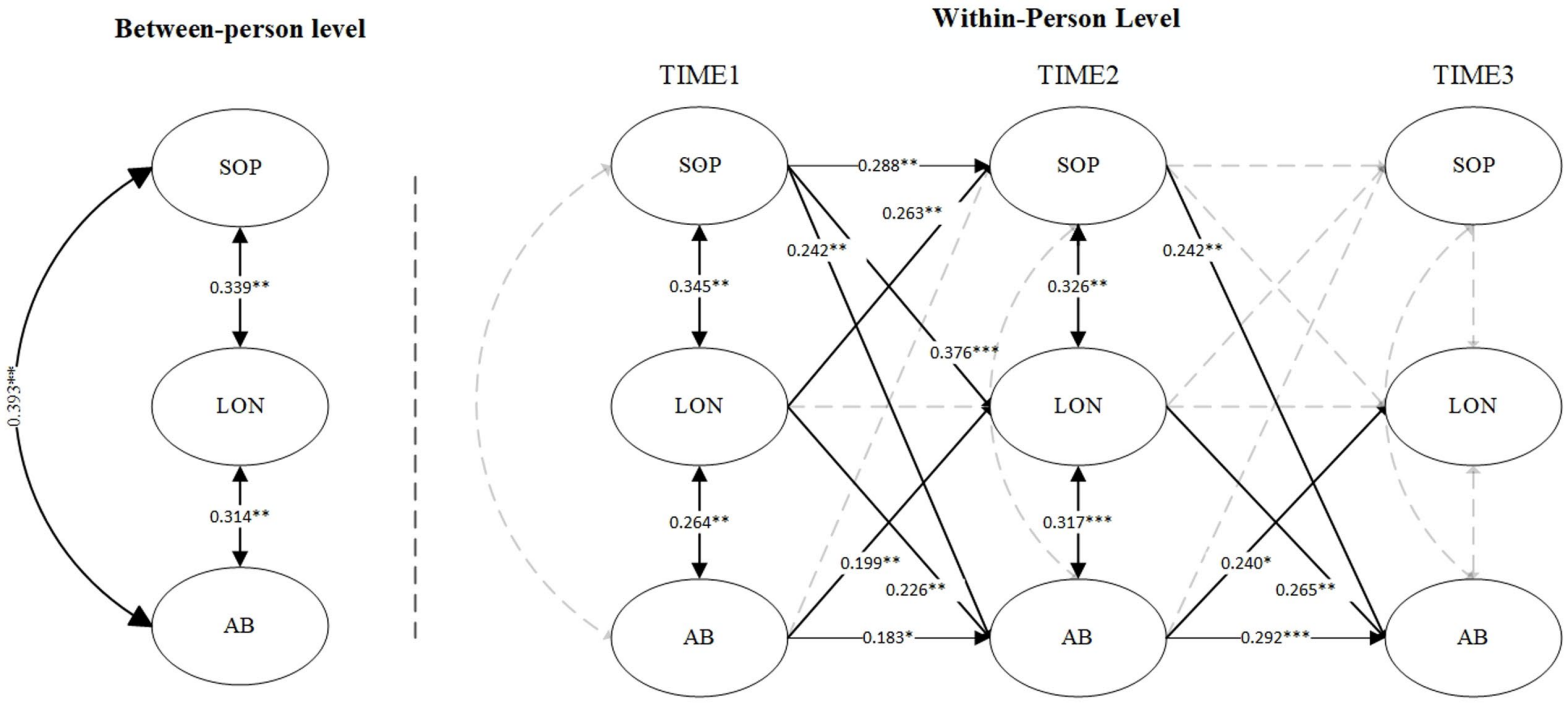
RI-CLPM for self-oriented perfectionism, loneliness, and athlete burnout (Model 2). SOP, Self-Oriented Perfectionism; LON, Loneliness; AB, Athlete burnout. Solid lines represent the significant paths, dashed lines represent the non-significant paths. *p < 0.05, **p < 0.01, ***p < 0.001.

Figure 2 presents the RI-CLPM results for SOP, loneliness, and athlete burnout. At the between-person level, SOP, loneliness, and athlete burnout demonstrated significant positive associations. At the within-person level, the pathway from SOP (T1) through loneliness (T2) to athlete burnout (T3) was significant (indirect effect = 0.099, 95% CI = 0.033–0.209) (see Table 4). This indirect effect (0.099) represents a small-to-moderate effect size, and the confidence interval (0.033–0.209) that excludes zero confirms a reliable mediating pathway with practical significance for sport psychology interventions. However, the reverse pathways were not significant. These findings collectively demonstrate that SOP exerts its influence on athlete burnout both directly and indirectly through loneliness, with effect sizes that are methodologically robust and practically meaningful enough to guide evidence-based interventions targeting perfectionism and social isolation as key mechanisms for burnout prevention.

**Table 4:** Indirect paths between self-oriented perfectionism, loneliness, and athlete burnout for the final RI-CLPM.

Indirect effects	RI-CLPM			
	β	SE	Bootstrapped 95% CI	
			Lower	Upper
SOPT1 → LONT2 → AB T3	0.099*	0.043	0.033	0.209
ABT1 → LONT2 → SOP T3	−0.036	0.029	−0.119	0.002

SOP, self-oriented perfectionism; LON, loneliness; AB, athlete burnout; CI, confidence interval; SE, standard error; *p < 0.05, **p < 0.01, ***p < 0.001.

### 4.4 Auxiliary analyses

We constructed a Cross-Lagged Panel Model (CLPM) for auxiliary analysis (see Supplementary material). The CLPM results indicated that SOP exhibited a unidirectional positive association with athlete burnout. Additionally, the pathway from SOP (T1) through loneliness (T2) to athlete burnout (T3) was significant, whereas the reverse pathways were not significant.

### 4.5 Gender invariance analyses

Multi-group modeling was employed to examine gender differences. The comparison between the gender-constrained model and the unconstrained model did not reveal statistically significant differences in model fit (SOP, professional burnout, and loneliness RI-CLPM: S-BΔ, χ^2^ = 17.853, Δdf = 12, *p* > 0.05; SOP and athlete burnout RI-CLPM: S-BΔ, χ^2^ = 10.979, Δdf = 8, *p* > 0.05). These findings indicate that the results do not differ by gender.

## 5 Discussion

The current research findings concerning the relationship between SOP and athlete burnout are complex and often contradictory. To clarify these discrepancies, the present study employed a longitudinal design, tracking three waves of athlete data. By utilizing the RI-CLPM, this study distinguished between stable between-person trait differences and within-person fluctuations, providing multi-level longitudinal evidence for the causal relationship between SOP and athlete burnout.

### 5.1 The relationship between self-oriented perfectionism and athlete burnout

In the RI-CLPM analysis, the between-person results indicated a positive association between SOP and athlete burnout. After controlling for the trait effects of various variables, the within-person results showed that SOP at T1 and T2 significantly predicted athlete burnout levels at T2 and T3, respectively. This suggests that athletes with higher levels of SOP at a given time point are more likely to experience burnout, which aligns with previous research (Hill et al., 2008; Hill and Appleton, 2011; Luo et al., 2016; Martin et al., 2022; Minichiello et al., 2024; Dišlere et al., 2025). These studies suggest that when SOP athletes fail to meet their high standards, they may be prone to developing burnout. A meta-analysis further indicates that multidimensional perfectionism consistently demonstrates significant associations with burnout, whereby the high standard demands characteristic of self-oriented perfectionism create chronic psychological stress (Hill and Curran, 2016). Jowett et al. (2016) examined the interaction between different dimensions of perfectionism and motivational factors, noting that when self-oriented components combine with controlled motivational regulation, burnout may emerge even in adolescent sport contexts. Similarly, Madigan et al. (2016), based on a two-wave longitudinal study, found that changes in personal standards (a core indicator of SOP) were significantly correlated with changes in burnout symptoms over several months, further validating the predictive role of SOP in burnout development. According to the cognitive-affective stress model, burnout is an emotional and behavioral syndrome that emerges when individuals struggle to cope with high external pressure and imbalanced self-evaluation (Chang et al., 2017; Lin et al., 2022). Additionally, athletes with high levels of perfectionistic concerns often experience higher levels of stress during sports because of their relentless pursuit of unattainable goals and intense fear of failure (Kang and Gong, 2024). This perceived stress is a key pathway through which perfectionistic concerns increase athletes’ vulnerability to burnout (Olsson et al., 2022a).

Another noteworthy finding is that the between-person results indicated that athlete burnout levels at T1 and T2 did not predict SOP levels at T2 and T3. This suggests that the reverse causal relationship between SOP and athlete burnout is not established. A possible explanation is that perfectionism, as a personality trait, often requires long-term, deep environmental interaction or systematic intervention to change (Stricker et al., 2019) rather than being influenced by short-term or episodic events (e.g., a period of burnout). According to the trait–state model in personality psychology, temporary psychological states typically do not reshape one’s core personality traits unless those states are extreme and prolonged (Anusic and Schimmack, 2016; Di Sarno et al., 2023; Kenny and Zautra, 1995). In contrast, personality traits tend to predispose individuals toward certain states or outcomes. In a three-month longitudinal study of adolescent athletes, perfectionistic concerns predicted increases in burnout, while perfectionistic strivings predicted decreases in burnout. However, the study found no evidence that levels of burnout subsequently influenced perfectionism—indicating a unidirectional longitudinal relationship, which is consistent with the findings of the present study. Therefore, even if an athlete’s attitude, enthusiasm, or behavior toward sports changes, it does not necessarily imply a departure from the underlying perfectionistic mindset or personality tendency. Moreover, the time lag in the present study may have been too short to capture the cumulative effects of burnout on personality-related traits such as perfectionism. Developmental psychologists have suggested that meaningful changes in personality traits typically unfold over the course of years rather than within a span of just a few months (Jackson and Wright, 2024). If athlete burnout does influence perfectionism, such effects may only emerge when athletes experience repeated or prolonged episodes of burnout over a longer developmental period, which may subsequently reshape their self-expectations. Therefore, future research should consider adopting a longer-term perspective—such as tracking cycles of burnout and recovery across multiple seasons or years—to examine whether burnout can prospectively predict changes in perfectionism.

These findings offer a new perspective on the current debate in this field. Compared to previous cross-sectional studies (Hill et al., 2008; Kang and Gong, 2024; Xu et al., 2024), this study provides stronger evidence through longitudinal data analysis, supporting the potential influence of SOP on the development of athlete burnout. Meanwhile, these findings hold important practical implications for coaches and youth sports organizations. Young athletes who set excessively high personal standards or exhibit self-critical tendencies should be considered at elevated risk for sport burnout and related mental health concerns. Coaches and sports organizations should implement regular psychological assessments and monitoring procedures to identify such risks early. Proactive intervention strategies—such as appropriately adjusting training loads, offering support and encouragement in the face of setbacks, and fostering team cohesion and robust social support systems—are essential in mitigating these risks. Moreover, coaches should recognize that empathy and understanding are just as critical as the pursuit of high performance standards. In both training and athlete management, efforts should be made to cultivate an environment that emphasizes growth and development rather than purely outcome-driven goals. Reinforcing effort, improvement, and team collaboration—rather than punishing imperfect performances—can effectively reduce maladaptive tendencies such as overtraining, excessive self-criticism, and social withdrawal. Ultimately, such an approach supports the long-term psychological well-being and sustained athletic development of youth athletes.

### 5.2 The indirect predictive role of loneliness

The between-person results of the RI-CLPM analysis indicated that loneliness was positively associated with both SOP and athlete burnout. At the within-person level, SOP emerged as an indirect predictor of athlete burnout via loneliness, whereas the reverse predictive path was not statistically significant. These findings are consistent with previous research. For example, DeFreese and Smith (2014) employed a longitudinal approach to assess the impact of social support and negative social interactions on athletes’ mental health over a competitive season. Their results demonstrated that maladaptive social interactions were associated with professional burnout. Chen et al. (2024) demonstrated that loneliness serves as a critical indicator of social disconnection, mediating the relationship between interpersonal perfectionism and psychological distress. Athletes with high SOP may establish excessively high personal standards and inadvertently impose these expectations on others, thereby compromising their ability to form meaningful interpersonal relationships (Gano-Overway and Peterson, 2023). Similarly, research by Chang et al. (2011) demonstrates that SOP, characterized by self-criticism and unrealistic high standards, impedes authentic interpersonal connections, thereby exacerbating loneliness. Thus, SOP may prompt individuals to engage in behaviors that hinder social connections, further reinforcing their isolated state and perpetuating loneliness over time (Dang et al., 2020). Furthermore, Gustafsson et al. (2017) emphasize that negative social experiences, including loneliness, constitute important risk factors for athlete burnout. Jackman et al.'s (2024) review also indicates that in sport contexts, loneliness is not only associated with impaired mental health but may also lead to negative motivational outcomes and maladaptive behaviors, with burnout being considered a particularly prominent risk consequence.

These findings provide practical guidance for psychological interventions aimed at athletes. Coaches can design targeted interventions to help athletes establish stronger interpersonal connections by enhancing team cohesion, strengthening social support networks, and promoting a sense of belonging within the team. Such efforts may help prevent or alleviate burnout. This finding carries significant implications for athletes. Athletes should recognize that an excessive internal drive for perfection may inadvertently contribute to social withdrawal or isolation, thereby intensifying feelings of loneliness. Consequently, it is particularly important to establish and maintain healthy social relationships outside of intense training and competition. This includes not only positive interactions with teammates but also meaningful communication with family members and friends outside the athletic domain. A diverse and stable social support network serves as a critical psychological buffer against loneliness and plays a preventative role in athletic burnout. Such support systems are conducive to the overall well-being and sustainable development of athletes. In terms of individualized intervention, it is recommended to tailor cognitive-behavioral strategies for athletes exhibiting pronounced perfectionistic tendencies. A growing body of research has demonstrated the effectiveness of cognitive-behavioral therapy (CBT) techniques—such as cognitive restructuring of perfectionistic thought patterns, mindfulness training, and stress management skills—in helping individuals adjust their mindset and coping mechanisms (Demir and Ercan, 2022; Nakao et al., 2021). In athletic practice, coaches can collaborate with athletes to identify and challenge irrational beliefs (e.g., “If I am not perfect, I am a failure”) and replace them with more realistic and self-compassionate perspectives, thereby reducing tendencies toward self-criticism. Moreover, the integration of mindfulness practices and relaxation techniques can enhance athletes’ attentional focus and mitigate performance anxiety associated with perfectionistic pressure, ultimately fostering psychological resilience and performance consistency.

### 5.3 Limitations

First, the data collection in this study relied on athletes’ self-reports, which may introduce common method bias due to the single-source nature of the data. This methodological limitation could inflate the observed correlations between variables. This is particularly relevant when assessing relatively sensitive psychological constructs such as perfectionism and loneliness, where social desirability bias may compromise the authenticity of the responses. Participants may be inclined to provide socially acceptable answers rather than candid reflections. To mitigate this limitation, future research could incorporate qualitative methods such as interviews to further validate and enrich the findings of the present study. Second, the sample in this study was drawn from China, where the meaning and expression of loneliness may be profoundly shaped by cultural context. Within the framework of Chinese collectivist culture, athletes’ feelings of loneliness are more likely to stem from a perceived lack of group belonging, unmet collective expectations, or marginalization within key relational networks (e.g., coach-athlete relationships, peer dynamics), rather than from thwarted autonomy or self-actualization, which are more commonly emphasized in Western individualistic cultures. For instance, when athletes feel disconnected from their teams, unable to contribute to collective success, or alienated in interpersonal relationships, experiences of loneliness may become particularly salient. Therefore, future cross-cultural studies are warranted to include athlete samples from diverse cultural backgrounds to assess the generalizability and cultural specificity of the current findings. Finally, this study focused on the predictive role of self-oriented perfectionism in athlete burnout, without examining other important dimensions of perfectionism, such as socially prescribed perfectionism (SPP). Future longitudinal studies should explore the distinct mechanisms through which different facets of perfectionism contribute to the development of burnout. Such work would deepen our understanding of psychological risk factors affecting athlete mental health.

## 6 Conclusion

This study revealed a longitudinal causal relationship between SOP and athlete burnout, indicating that increases in SOP significantly predicted higher levels of burnout, whereas athlete burnout did not predict increases in SOP. Moreover, the study found that SOP can indirectly predict athlete burnout through loneliness, suggesting that SOP contributes to burnout by exacerbating athletes’ feelings of loneliness. Based on these findings, several strategies could be implemented by management to reduce the risk of occupational burnout among athletes with high levels of SOP. For instance, enhancing social support through team-building activities, providing greater coach support, or developing cognitive-behavioral interventions aimed at reducing self-criticism, such as Acceptance and Commitment Therapy (ACT), may prove beneficial. Additionally, athletes themselves could proactively seek social support or employ strategies such as mindfulness and cognitive-behavioral therapy (CBT) to enhance psychological resilience and mitigate negative effects.

## Data Availability

The original contributions presented in the study are included in the article/Supplementary material, and further inquiries can be directed to the corresponding authors.
