# Supplementary material for: The relationship between self-oriented perfectionism and athlete burnout: a longitudinal study
Source: Front Psychol. 2025 Oct 16;16:1656816. doi: 10.3389/fpsyg.2025.1656816 (PMC12572936; doi:10.3389/fpsyg.2025.1656816)
Supplement: Supplementary file 1 [file Supplementary_file_1.docx]

Appendix： The Results of CPLMs Analyses

We constructed traditional Cross-Lagged Panel Models (CLPM) to examine the longitudinal associations among Self-Oriented Perfectionism, Loneliness, and Athlete burnout under conditions where both between-person and within-person confounding effects coexist. In all CLPMs, participants' gender, age and professional tenure were entered as time-invariant covariates across three time points. The optimal model was selected through a systematic model comparison procedure where longitudinal structural parameters, including both autoregressive paths and cross-lagged paths, were subjected to stepwise time-invariance constraints.

**Table S1** Model fit of CLPMs.

| Models | χ*^2^(df)* | CFI | SRMR | RMSEA [90% CI] | ∆CFI | ∆RMSEA |
| --- | --- | --- | --- | --- | --- | --- |
| Model 1 |  |  |  |  |  |  |
| **M1a** | **127.913 (27)** | **0950** | **0.050** | **0.091 [0.075 0.107]** | **-** | **-** |
| M1b | 151.608 (31) | 0.941 | 0.054 | 0.093 [0.078 0.108] | 0.009 | 0.002 |
| M1c | 155.564 (36) | 0.941 | 0.057 | 0.086 [0.100 0.124] | 0.009 | 0.005 |
| Model 2 |  |  |  |  |  |  |
| **M2a** | **63.448 (16)** | **0.958** | **0.044** | **0.081 [0.061 0.102]** | **-** | **-** |
| M2b | 70.387 (18) | 0.954 | 0.052 | 0.080 [0.061 0.100] | 0.004 | 0.001 |
| M2c | 65.435 (18) | 0.958 | 0.045 | 0.076[0.057 0.097] | 0.000 | 0.005 |

Note: CLPM = Cross-Lagged Panel Model; M1a / M2a = Fully unconstrained CLPM; M1b / M2b = CLPM with time invariance constrains on the autoregressive stabilities; M1c / M2c = CLPM with time invariance constrains on the cross-lagged effects. CFI = Comparative Fit Index; RMSEA = Root Mean Square Error of Approximation; CI = Confidence Interval; SRMR = Standardized Root Mean Square Residual.


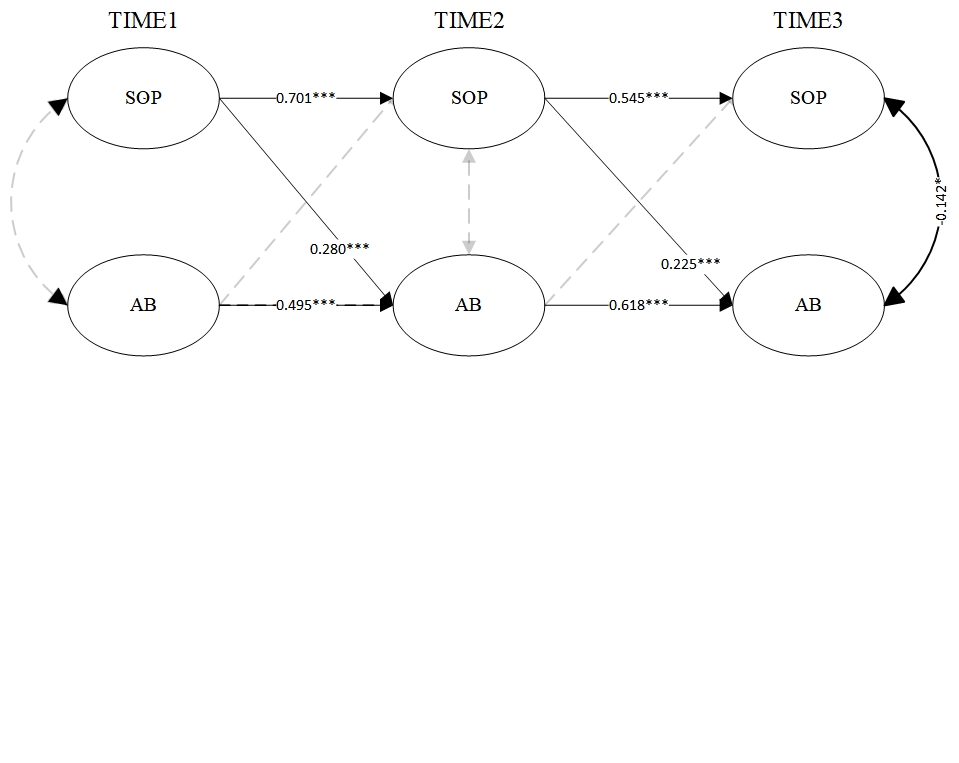


**Fig 1**. CLPM for Self-Oriented Perfectionism and Athlete Burnout (Model 1).

Note. SOP=Self-Oriented Perfectionism, AB=Athlete burnout. Solid lines represent the significant paths, dashed lines represent the non-significant paths*p < .05, **p < .01, *** p < .001.


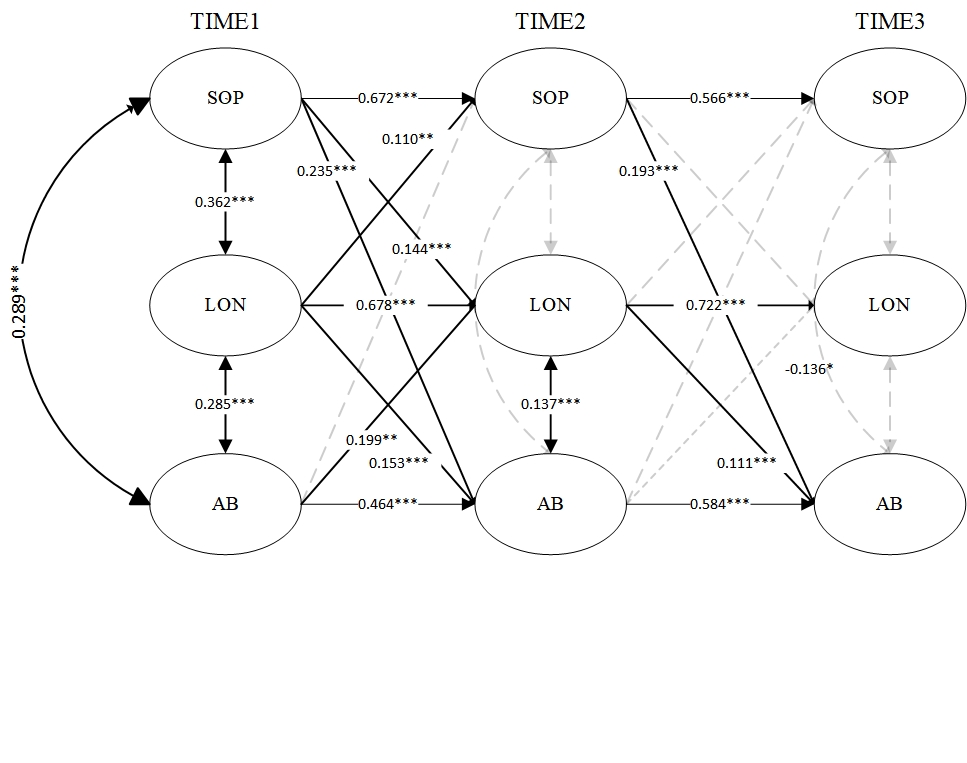


**Fig 2**. CLPM for Self-Oriented Perfectionism, Loneliness, and Athlete Burnout .

Note. SOP=Self-Oriented Perfectionism, LON= Loneliness, AB=Athlete burnout. Solid lines represent the significant paths, dashed lines represent the non-significant paths. *p < .05, **p < .01, *** p < .001.

**Table S2** Indirect paths between AU, PSC, and AB for the final RI-CLPM (Standardized Coefficients).

| Indirect Effects | RI-CLPM | | | |
| --- | --- | --- | --- | --- |
|  | $\beta$ | SE | Bootstrapped  95% CI | |
|  |  |  | Lower | Upper |
| SOPT1 → LON T2 → AB T3 | 0.006** | 0.002 | 0.002 | 0.010 |
| ABT1 → LONT2 → SOP T3 | 0.000 | 0.001 | -0.001 | 0.002 |

Note. SOP=Self-Oriented Perfectionism, LON= Loneliness, AB=Athlete burnout. *p < .05, **p < .01, *** p < .001.
